# Bioactivity of Medicinal Plants and Extracts

**DOI:** 10.3390/biology10070634

**Published:** 2021-07-08

**Authors:** Francisco Les, Guillermo Cásedas, Víctor López

**Affiliations:** 1 Department of Pharmacy, Faculty of Health Sciences, Universidad San Jorge, 50830 Zaragoza, Spain; gcasedas@usj.es (G.C.); ilopez@usj.es (V.L.); 2 Instituto Agroalimentario de Aragón, IA2, Universidad de Zaragoza-CITA, 50059 Zaragoza, Spain

Nature is an inexhaustible source of bioactive compounds and products with interesting medicinal properties and technological applications. Although natural products can be found in plants, animals, microorganisms and minerals, the vast majority of them come from plants [1].

Since the beginning of the ages, plants have produced a great variety of molecules through different biosynthetic routes. Some of them are considered essential for the normal performance and development of the plant, such as carbohydrates, lipids and proteins; this aggregate is called primary metabolites [2].

The biochemical pathways also lead to the production of relatively small molecules known as secondary metabolites. These secondary metabolites do not seem essential for plant development. However, science has demonstrated that secondary metabolites have important functions in plants, for instance, defence against ultraviolet radiation exposure; struggling against infections caused by viruses, fungi, bacteria and phytopathogens; or keeping herbivores away. These secondary metabolites are the most interesting in therapeutics and belong to three large groups known as polyphenols, terpenes and alkaloids [3].

Natural products have constituted the origin of pharmacology and therapeutics. Early on, they were used as medicinal plants or preparations, and later as isolated molecules or phytochemically characterized extracts. Plants are still a source in nature for obtaining and isolating molecules with pharmacological applications (drug discovery), but can also be used as herbal medicinal products in traditional or complementary medicine. In addition, the WHO has launched a Traditional Medicine Strategy (2014–2023), including herbal medicines as medicinal therapies, with the aim of ensuring the quality, safety, proper use and effectiveness of traditional medicines, among other objectives [4].

More recently, natural products have continued to enter clinical trials or to provide leads for compounds that have entered clinical trials, particularly as anticancer and antimicrobial agents. Further, research in natural products has shown many advantages [5,6]:

— Nature presents a chemical diversity that is practically immeasurable by human mind.
— Natural compounds, by virtue of being biosynthesized by living organisms, are expected to be “drug-like” because they have already had to interact with enzymes, receptors and biological signalling pathways.
— Bioactive compounds that come from species used in traditional medicine have a greater probability of success because they are already being used for therapeutic purposes.

Throughout history, human have used plants for therapeutic purposes; the development of synthetic and organic chemistry allowed herbal medicines to be replaced by isolated molecules provided by the pharmaceutical industry, even though approximately 50% of them are not completely synthetic and have a natural origin [7,8,9]. It is also important to note that research on natural products has increased exponentially in recent years, but the percentage of new approved drugs that have a natural origin has decreased. This fact has been caused by different factors, such as aspects of intellectual property, respect for biodiversity, accessibility to living organisms or the amount of active substance available in nature [10,11].

In addition to their nutritional, industrial, ecological and environmental value, plants have played (and still continue to play) a crucial role in medicine and pharmacy [12].
